# Facile Preparation Method of TiO_2_/Activated Carbon for Photocatalytic Degradation of Methylene Blue

**DOI:** 10.3390/mi15060714

**Published:** 2024-05-29

**Authors:** Phuoc Huu Le, Tran Thi Thuy Vy, Vo Van Thanh, Duong Hoang Hieu, Quang-Thinh Tran, Ngoc-Van Thi Nguyen, Ngo Ngoc Uyen, Nguyen Thi Thu Tram, Nguyen Chi Toan, Ly Tho Xuan, Le Thi Cam Tuyen, Nguyen Trung Kien, Yu-Min Hu, Sheng-Rui Jian

**Affiliations:** 1 Center for Plasma and Thin Film Technologies, Ming Chi University of Technology, New Taipei City 24301, Taiwan; 2 International Ph.D. Program in Plasma and Thin Film Technology, Ming Chi University of Technology, New Taipei City 24301, Taiwan; 3 Faculty of Basic Sciences, Can Tho University of Medicine and Pharmacy, 179 Nguyen Van Cu Street, Can Tho City 900000, Vietnam; nnuyen@ctump.edu.vn (N.N.U.); ntttram@ctump.edu.vn (N.T.T.T.); 4 Faculty of Pharmacy, Can Tho University of Medicine and Pharmacy, 179 Nguyen Van Cu Street, Can Tho City 900000, Vietnam; 1953030090@student.ctump.edu.vn (T.T.T.V.); vovanthanhct2020@gmail.com (V.V.T.); 1953030015@student.ctump.edu.vn (D.H.H.); 2053030101@student.ctump.edu.vn (Q.-T.T.); ntnvan@ctump.edu.vn (N.-V.T.N.); 5 Faculty of Pharmacy and Nursing, Tay Do University, 68 Tran Chien Street, Can Tho City 900000, Vietnam; nctoan@tdu.edu.vn; 6 Department of Materials Science and Engineering, National Taiwan University Science and Technology, Taipei City 106335, Taiwan; lyxuan.141@gmail.com; 7 Faculty of Chemical Engineering, Can Tho University, 3/2 Street, Ninh Kieu District, Can Tho City 900000, Vietnam; ltctuyen@ctu.edu.vn; 8 Faculty of Medicine, Can Tho University of Medicine and Pharmacy, 179 Nguyen Van Cu Street, Can Tho City 900000, Vietnam; ntkien@ctump.edu.vn; 9 Department of Applied Physics, National University of Kaohsiung, Kaohsiung 81148, Taiwan; ymhu@nuk.edu.tw; 10 Department of Materials Science and Engineering, I-Shou University, Kaohsiung 84001, Taiwan

**Keywords:** TiO_2_/activated carbon, mass mixing ratio, photocatalyst, methylene blue

## Abstract

The development of nanocomposite photocatalysts with high photocatalytic activity, cost-effectiveness, a simple preparation process, and scalability for practical applications is of great interest. In this study, nanocomposites of TiO_2_ Degussa P25 nanoparticles/activated carbon (TiO_2_/AC) were prepared at various mass ratios of (4:1), (3:2), (2:3), and (1:4) by a facile process involving manual mechanical pounding, ultrasonic-assisted mixing in an ethanol solution, paper filtration, and mild thermal annealing. The characterization methods included XRD, SEM-EDS, Raman, FTIR, XPS, and UV-Vis spectroscopies. The effects of TiO_2_/AC mass ratios on the structural, morphological, and photocatalytic properties were systematically studied in comparison with bare TiO_2_ and bare AC. TiO_2_ nanoparticles exhibited dominant anatase and minor rutile phases and a crystallite size of approximately 21 nm, while AC had XRD peaks of graphite and carbon and a crystallite size of 49 nm. The composites exhibited tight decoration of TiO_2_ nanoparticles on micron-/submicron AC particles, and uniform TiO_2_/AC composites were obtained, as evidenced by the uniform distribution of Ti, O, and C in an EDS mapping. Moreover, Raman spectra show the typical vibration modes of anatase TiO_2_ (e.g., E_1g_^(1)^, B_1g_^(1)^, E_g_^(3)^) and carbon materials with D and G bands. The TiO_2_/AC with (4:1), (3:2), and (2:3) possessed higher reaction rate constants (*k*) in photocatalytic degradation of methylene blue (MB) than that of either TiO_2_ or AC. Among the investigated materials, TiO_2_/AC = 4:1 achieved the highest photocatalytic activity with a high *k* of 55.2 × 10^−3^ min^−1^ and an MB removal efficiency of 96.6% after 30 min of treatment under UV-Vis irradiation (120 mW/cm^2^). The enhanced photocatalytic activity for TiO_2_/AC is due to the synergistic effect of the high adsorption capability of AC and the high photocatalytic activity of TiO_2_. Furthermore, TiO_2_/AC promotes the separation of photoexcited electron/hole (e^−^/h^+^) pairs to reduce their recombination rate and thus enhance photocatalytic activity. The optimal TiO_2_/AC composite with a mass ratio of 4/1 is suggested for treating industrial or household wastewater with organic pollutants.

## 1. Introduction

Water is an essential resource for the growth of the natural environment on earth and people. Water pollution with organic dyes, heavy metals, and other contaminants (i.e., pesticides, steroid hormones, and antibiotics) is a current environmental issue [1]. Synthetic organic dyes are omnipresent in many application areas of the textile, tannery, cosmetic and food industries, and medicine [1]. The dying process in the textile and tannery industries can release a vast majority of organic dyes due to the low efficiency of the dying process, with approximately 15% of dye lost [1,2]. Organic dye pollution in the aquatic environment poses a threat to animal or human health and causes adverse effects on aquatic biota and water ecosystems [1,3]. In 1995, M.R. Hoffmann et al. [4] presented the underlying principles governing semiconductor photocatalysis and its potential applications as an environmental control technology, and this field has been greatly developed over the years [5,6].

Titanium dioxide (TiO_2_) is widely recognized as one of the most practical and prevalent photocatalysts due to its remarkable photocatalytic activity in the ultraviolet (UV) region, cost-effectiveness, chemical stability, abundance, and non-toxic nature [5]. However, TiO_2_ faces two primary limitations. Firstly, it remains inactive in the visible (Vis) range owing to its wide band gap semiconductor, with Eg = 3.2 eV for anatase and 3.0 eV for rutile [7,8]. Secondly, TiO_2_ exhibits rapid recombination of photoexcited carriers (electrons and holes) [9], prompting considerable interest in heterocatalyst and composite approaches.

TiO_2_ P25 (a powder-form photocatalyst) offers relatively high photocatalytic degradation of organic dyes [10] and pharmaceuticals [10,11,12,13] and a high possibility of practical-scale application. However, TiO_2_ P25 use in the form of suspension (slurry) may pose an ecological risk to aquatic organisms [14], which should require an expensive filtration process to separate the suspension catalyst from the treated water. Therefore, many nanostructured TiO_2_ films developed on rigid substrates by the anodizing method (e.g., TiO_2_ nanotubes [15], TiO_2_ nanowires on TiO_2_ nanotube arrays [16,17,18]) have been studied for photocatalyst, solar energy conversion, and other applications [15]. In addition, nanoparticular TiO_2_ films can be synthesized successfully on various substrates (Si, quartz, or sapphire) using a gas-phase method of supersonic cluster beam deposition (SCBD) for studying the photodegradation of salicylic acid [19] and propane oxidation under 375 nm UV-LED illumination (8 mW/cm^2^) [20]. Also, Ag and Ag/TiO_2_ nanocomposite films were grown by SCBD to investigate the effects of structure morphology on the optical properties of the films [21]. Photocatalytic activity in film forms of TiO_2_ nanomaterials faces a big challenge in scaling up to the practical scale of water treatment. Another approach is that TiO_2_ nanomaterial is loaded on/in another bigger nano/micro-scale structure to form a heterostructure or a composite. Heterostructure and composite photocatalysts can offer activity enhancement due to some mechanisms related to manipulating the photoresponse region and the fate of the electron/hole pairs [8]. In this way, TiO_2_ has been impregnated on several porous supports such as silica, alumina, zeolite, and carbon materials [e.g., activated carbon (AC)] [22,23,24]. TiO_2_/AC has been found to possess higher photocatalytic activity than bare TiO_2,_ as it combines the good photocatalytic activity of TiO_2_ with the high surface area of AC [23,24,25,26,27,28,29,30]. In addition, TiO_2_ nanoparticles loaded in the AC immobilization can resolve the limitation of using TiO_2_ suspension, while AC is a very economical and useful adsorbent and is characterized by a high surface area, micro- to macro-pore structures, and a high degree of surface reactivity [31].

Many studies focused on developing TiO_2_/AC with structural, compositional, and surface functional modification, which usually requires the use of more specialized chemicals and laboratory equipment for the material synthesis via the sol-gel methods [23,24,28,29], hydrothermal and reflux methods [30], hydrothermal-impregnation-carbonization-activation processes [27], ultrasonic-assisted sol-gel treatment and solvothermal treatment combined with microwave-assisted heating [26], and hydrolytic precipitation of TiO_2_ from tetrabutylorthotitanate and following heat treatment [25]. Noticeably, a complicated preparation process of TiO_2_/AC can hinder the practical application at the household level, where non-specialized people will be the operators of their household water treatment tank or plant. In this study, we prepared TiO_2_ P25/AC using the two commercial raw materials by a facile method by mixing TiO_2_/AC mechanically at different mass ratios. Differing from the previous studies, the compositional weight portions of TiO_2_/AC were in some specific range, such as TiO_2_/(1–15 wt.%)-AC [30], TiO_2_/(5–75 wt.%)-AC prepared by the sol-gel method [23], while the weight ratios of TiO_2_/AC in this study were TiO_2_/AC = 5:0, TiO_2_/AC = 4:1, TiO_2_/AC = 3:2, TiO_2_/AC = 2:3, TiO_2_/AC = 1:4, and TiO_2_/AC = 0:5. It is worthy of mention that the mass ratio unit for the composite should be easier and more convenient for normal people to apply in practice when they implement TiO_2_/AC material into their household water treatment plant. Moreover, this study has two reference samples (i.e., TiO_2_, AC) to obtain insight into the role of either TiO_2_ or AC in the photocatalytic degradation of methylene blue, which is a typical organic dye and considered a good model for studying photocatalyst activity and process. This study provides the effect of mixing weight ratios of TiO_2_/AC on the morphological, structural, compositional, and photocatalytic properties of the composite.

## 2. Materials and Methods

### 2.1. Materials

**Figure 1:**
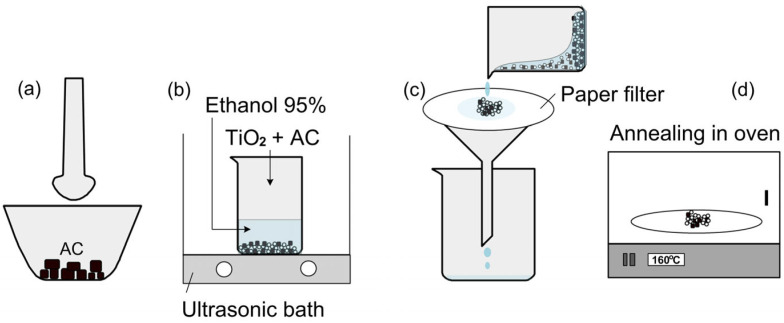
TiO_2_/AC preparation process: (**a**) Crushing TiO_2_-AC powder using a mortar and pestle, (**b**) Mixing the powder in 95% methanol under ultrasonic shaking, (**c**) Filtering the powder using a paper filter, (**d**) Annealing the powder in an oven at 160 °C.

Commercial Degussa TiO_2_ P25 was purchased from Merck (Darmstadt, Germany), and activated carbon (AC) was a product of Tra Bac Company, Tra Vinh City, Vietnam, which was made from coconut shell. First, TiO_2_ Degussa P25 was activated in a NaOH 5 M solution at room temperature for 100 min, then it was filtered and cleaned with deionized water before drying in an oven at 100 °C for 3 h. Then, AC (~5 g) was placed in a clean mortar, then crushed with a pestle for 30 min (Figure 1a). Next, AC was mixed with TiO_2_ nanoparticles (P25) at different weight ratios, while the total weight of the material (TiO_2_ + AC) was fixed at 0.5 g. Six types of samples were prepared, including S1 (TiO_2_), S2 (TiO_2_/AC = 4:1), S3 (TiO_2_/AC = 3:2), S4 (TiO_2_/AC = 2:3), S5 (AC/TiO_2_ = 1:4), and S6 (AC). Specifically, for example, we mixed 0.3 g TiO_2_ with 0.2 g AC to prepare the S3 sample. To obtain a uniform mixture and surface functionalize for the TiO_2_/AC composite, the 0.5 g powder was placed in a beaker filled with 10 mL of 95% ethanol under sonication for 30 min (Figure 1b). Next, the powder mixture was filtered using a paper filter (Figure 1c). Finally, the material was annealed in an oven at 160 °C for 3 h. The powder products were stored in glass containers for later use.

### 2.2. Photocatalytic Activity in Degradation MB of TiO_2_/AC

Photocatalysis was carried out by placing 10 mg of the material powder in a 30 mL MB (at an initial concentration of 10 mg/L; the catalyst dosage of 333.3 mg/L) beaker and irradiating with ultraviolet-visible (UV-VIS) radiation from a Xenon lamp (Prosper OptoElectronic Co., Ltd., Yilan County, Taiwan; power 100 W and power density of 120 mW/cm^2^). The temperature of all QXT reaction beakers was kept at 32–33 °C. After each given photocatalytic reaction time (15, 30, 45, or 60 min), 1 mL of MB solution was extracted for qualitative and quantitative study of the relative concentration of MB by UV-VIS spectrophotometer (Hitachi U-2900, Hitachinaka, Japan) through the characteristic absorption peak of MB at 655 nm.

### 2.3. Characterization Methods

The orientation and crystallinity of the materials were determined by X-ray diffraction (XRD, Bruker D2, Bruker, Billerica, MA, USA) using Cu K_α_ radiation (λ = 1.5406 Å) and θ–2θ configuration. The morphology of the samples was determined by a scanning electron microscope (SEM, Hitachi S-4800, Tokyo, Japan). The chemical composition was analyzed by X-ray energy dispersive spectroscopy (EDS, Oxford probe, Oxfordshire, UK) and integrated SEM (JEOL JSM-IT700HR, Tokyo, Japan). The Raman spectrum of a selected TiO_2_/AC was obtained using a Horiba Evolution (Labram HR model, Kyoto, Japan) spectrometer operated with an excitation wavelength of 514 nm. The FTIR-ATR spectra of TiO_2_ and selected TiO_2_/AC powders were acquired using the PerkinElmer FT-IR/NIR spectrometer (Spectrum Two^TM^, Perkin Elmer Inc., Waltham, MA, USA), which was equipped with a Universal ATR (attenuated total reflectance) sampling accessory containing a diamond/ZnSe crystal. The surface chemistry of TiO_2_/AC was analyzed using X-ray photoelectron spectroscopy (XPS, ThermoVG 350, Waltham, MA, USA) with a Mg Kα X-ray source (power of 300 W, photon energy of 1253.6 eV). Calibration of the XPS spectra was performed using the C1s peak at 284.7 eV. Curve fitting analysis was conducted using the XPSPEAK 4.1 software, assuming a Gaussian–Lorentzian peak shape and employing Shirley background subtraction.

## 3. Results and Discussion

**Figure 2:**
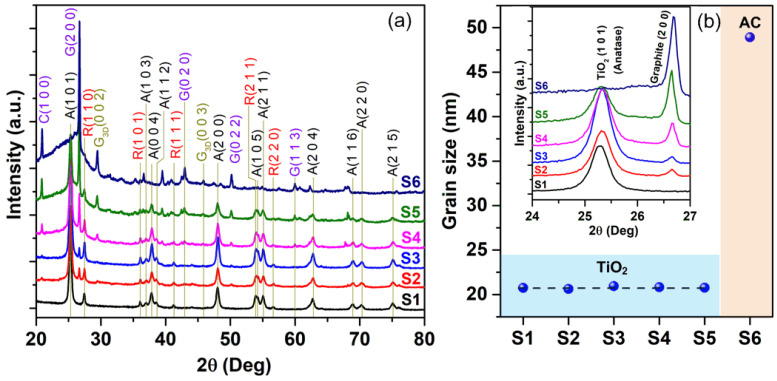
(**a**) X-ray diffraction patterns of TiO_2_, TiO_2_/AC composites, and AC. The symbol C (hkl) is the spectral peak of carbon (IMCSD #0013020); the symbols G (hkl) and G_3D_ (hkl) are the peaks of graphite (IMCSD #0000049) and graphite 3D structures (IMCSD #0013980), respectively; and A (hkl) and R (hkl) indicate the patterns of TiO_2_ anatase and TiO_2_ rutile phases, respectively. (**b**) The crystallite size of the materials; the inset is the zoom-in view of high-intensity characteristic peaks for TiO_2_ and AC.

Figure 2a presents the XRD pattern of TiO_2_ P25 nanoparticles (S1), TiO_2_/AC composites prepared at various mass ratios (S2–S5), and AC (S6). Generally, TiO_2_ had a dominant anatase phase characterized by diffraction peaks of A(101) at 25.3°, A(004) at 37.9°, A(200) at 48.2°, and A(204) at 62.8°, and a minor rutile phase with the observed peaks of R(110) at 27.5° and R(211) at 54.1°.

Meanwhile, the AC exhibited the diffraction peaks of graphite [e.g., G(002) at 26.6°, G(020) at 45.5°], carbon C(100) at 20.9°, and 3D carbon structures with peaks G_3D_(002) at 30.0°. The intensity of TiO_2_ and AC peaks varied reasonably with the evolution of TiO_2_/AC content (see Figure 2a). The inset of Figure 2b provides a zoom-in view of the TiO_2_(101) and G(200) peaks. The intensity of the G(200) peak increases monotonically with the rising AC content from S2 (TiO_2_/AC = 4:1) to S6 (TiO_2_/AC = 0:5). Reasonably, S1 (TiO_2_/AC = 5:0) does not exhibit the G(200) peak, whereas S6 lacks the TiO_2_(101) peak (Figure 2b inset). The dominant anatase phase over the rutile one for TiO_2_ Degussa P25 in this study is consistent with the results reported in ref. [32]. The XRD results are reasonable with the reported compositions of TiO_2_ P25, comprising 77.1% anatase, 15.9% rutile, and 7.0% amorphous TiO_2_ [33].

The crystallite size (*D*) in TiO_2_ P25 and AC was estimated using the Scherrer equation and TiO_2_ (101) and G(200) peaks, respectively. The Scherrer equation is given as *D* = 0.9*λ*/*β*cos*θ*, where *λ*, *β*, and *θ* are the X-ray wavelength, full width at half maximum of the diffraction peak, and Bragg diffraction angle, respectively [18,23,34]. The *D* value for TiO_2_ P25 in S1–S5 samples was a range of 20.6–21.0 nm, while the *D* of AC was 48.9 nm (Figure 2b). The *D* of TiO_2_ in this study is comparable with that of pristine TiO_2_ synthesized by the sol-gel method (D = 17.5 nm), smaller than the D values of 43.4–109.0 nm for the TiO_2_ annealed thermally at 200–500 °C for 120 min [28]. Noticeably, the crystallite size (48.9 nm) for the present AC is very close to the *D* of 50 nm for the commercial AC used in ref. [28].

**Figure 3:**
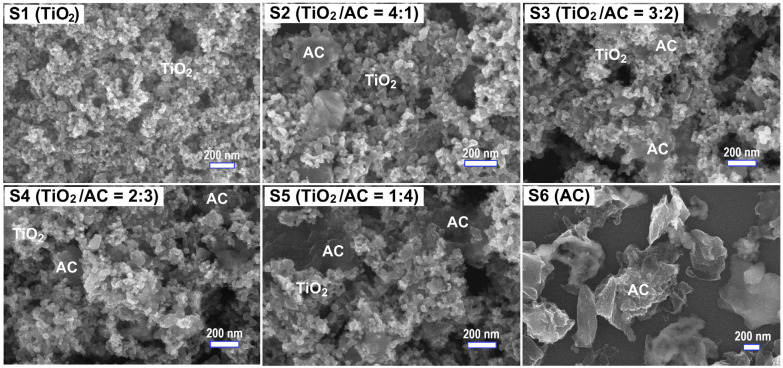
The typical scanning electron microscopy (SEM) images of TiO_2_ P25 (S1), TiO_2_/activated carbon (AC) composites prepared at various mass ratios (S2–S5), and AC (S6).

**Figure 4:**
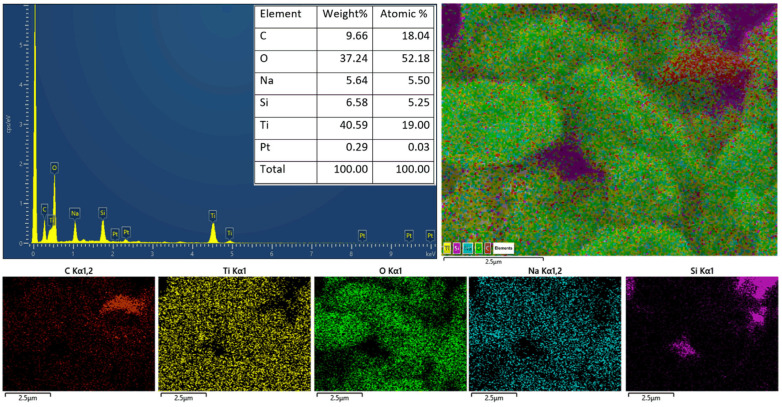
An EDS spectrum of a TiO_2_/AC prepared by dropping a DI water solution containing S4 powder on a clean Si substrate; and an EDS mapping of the S4 powder sample.

Figure 3 shows the surface morphology of the studied materials. TiO_2_ P25 exhibited uniform nanoparticles (NPs) with a size of 25–35 nm, while AC contained micron- and sub-micron particles. For TiO_2_/AC composites, TiO_2_ nanoparticles were well dispersed and decorated tightly with AC particles (Figure 3). The SEM images also reflect the contents of TiO_2_ and AC in the composites; e.g., TiO_2_ content decreases while AC content increases when we observe the SEM images from S2 to S5 (Figure 3). The elemental composition and distribution were illustrated by the EDS result (S4) in Figure 4. This composite has 18.04 at.% C, 52.18 at.% O, and 19.00 at.% Ti, which indicates a composition of AC/TiO_2_. Notably, small contents of Na (5.5 at.%), Si (5.25 at.%), and Pt (0.03 at.%) were observed due to the remaining after the surface activation process for P25 using NaOH 5 M, the Si substrate, and the Pt coating for taking SEM images, respectively. In addition, the EDS mapping in Figure 4 suggests a uniform elemental distribution, uniform decoration of TiO_2_ on AC, and possibly loading of TiO_2_ NPs inside the micro-pores and micro-channels of AC. The SEM and EDS results indicate the tight binding between TiO_2_ and AC that should support carrier transport for enhancing photocatalytic activity.

**Figure 5:**
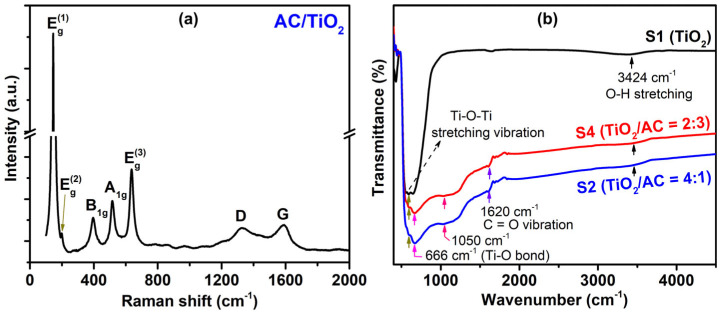
(**a**) A typical Raman spectrum of AC/TiO_2_. (**b**) FTIR spectra of TiO_2_ (S1), TiO_2_/AC (S2) and TiO_2_/AC (S4).

To gain insight into the crystalline structure of TiO_2_/AC composites, a typical Raman spectrum of TiO_2_/AC was examined. In Figure 5a, the spectrum exhibited characteristic peaks of the TiO_2_ predominant anatase phase at 146 cm^−1^ (E_g_^(1)^), 200 cm^−1^ (E_g_^(2)^), 396 cm^−1^ (B_1g_), 516 cm^−1^ (A_1g_), and 635 cm^−1^ (E_g_) as well as two graphite carbon peaks at 1327 cm^−1^ (D-band) and 1588 cm^−1^ (G-band) (Figure 5a). The D-band is associated with asymmetric lattices and bond-angle disorders in graphitic structures [27], while the G-band comes from the doubly-degenerate iTO and LO phonons with E_2g_ symmetry at the Brillouin zone center [35]. This Raman result for TiO_2_/AC agreed well with the Raman result for TiO_2_/AC in ref. [27].

To explore the interaction between TiO_2_ and AC, we recorded the FTIR spectra of TiO_2_ (S1) and TiO_2_/AC (S2, S4). As depicted in Figure 5b, the band observed in the range of 500–700 cm^−1^ corresponds to the FTIR band of TiO_2_, indicative of the Ti-O-Ti stretching vibration at approximately 593 cm^−1^ and the Ti-O bond at around 666 cm^−1^ [36]. Notably, all TiO_2_ and TiO_2_/AC samples exhibited a minor peak at approximately 3424 cm^−1^, attributed to the O–H stretching of hydroxyl groups resulting from moisture adsorption [36]. Moreover, the FTIR spectra of TiO_2_/AC materials revealed additional peaks at approximately 1050 cm^−1^ and around 1620 cm^−1^. These peaks are attributed to the ring vibration in aromatic compounds, primarily present in carbonaceous materials such as AC, and the C=O vibration, respectively [36].

**Figure 6:**
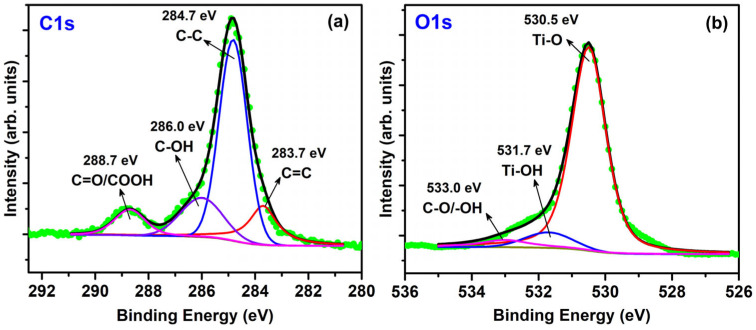
XPS spectra of a TiO_2_/AC = 4/1 (S2) for C1s (**a**) and O1s (**b**).

XPS spectra analysis of TiO_2_/AC (S2) was conducted to examine the chemical composition and bonding environment within TiO_2_/AC nanomaterials. In Figure 6a, the C1s spectrum can be deconvoluted into four Lorentzian-Gaussian peaks. The primary peak observed at 284.7 eV corresponds to C–C bonds, while three minor peaks appear at 283.7 eV for C=C bonds, 286.0 eV for C-OH due to –OH groups chemisorbed on the surface of TiO_2_, and 288.7 eV for C=O/COOH bonds [26]. This finding is consistent with previous studies on TiO_2_/AC [26] and TiO_2_-graphene systems [37]. In Figure 6b, the O1s spectrum is well-fitted with three peaks. Notably, a dominant peak at 530.5 eV corresponds to oxygen within the crystal lattice of TiO_2_, while two additional peaks at 531.7 and 533.0 eV are associated with Ti-OH and C-O/-OH, respectively. The present O1s spectrum result agrees well with that of TiO_2_/AC [26].

**Figure 7:**
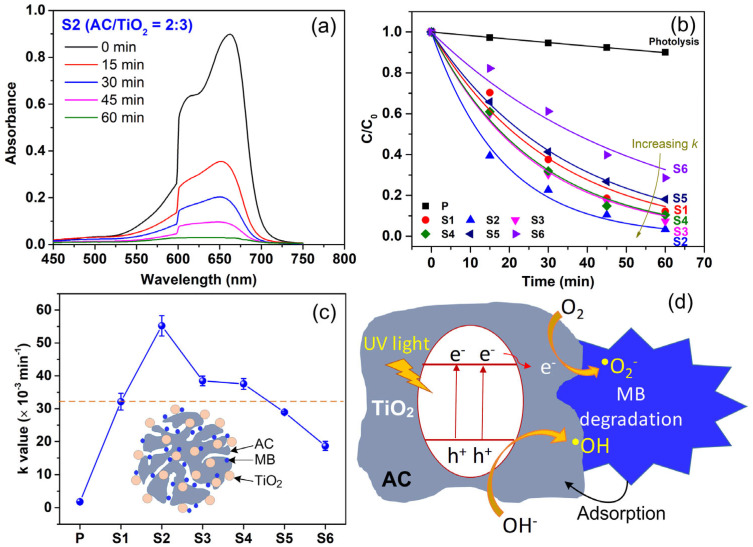
(**a**) Typical photodegradation of methylene blue (MB) using AC/TiO_2_ materials (e.g., S2 sample case). (**b**) Photocatalytic degradation of MB by AC/TiO_2_ materials prepared at various mass ratios under the UV-Vis irradiation of Xenon lamp (120 mW/cm^2^). (**c**) The pseudo-first-order kinetics constant k of photolysis (P) and photocatalytic reaction using AC/TiO_2_ (S1–S6); Inset in (**c**) shows a schematic of porous AC/TiO_2_ in MB solution. (**d**) A schematic diagram for the photocatalytic degradation mechanism of MB using AC/TiO_2_ composite.

The photocatalytic activities of TiO_2_, AC, and TiO_2_/AC with different mixing mass ratios were studied by monitoring the photodegradation kinetics of MB (an organic substance with popular use as an organic pollutant model) under UV-Vis irradiation (120 mW/cm^2^). Figure 7a shows the evolution of the MB absorption peak (at ~655 nm) over the photocatalytic reaction time (t) using the TiO_2_/AC (S2). The peak intensity (associated with MB concentration) decreased with *t,* and this behavior is true for the other photocatalysts in this study (S1, S3, S4, S5, S6). The photodegradation kinetics by photolysis and photocatalytic processes using the materials are shown in Figure 7b, which obeys the Langmuir–Hinshelwood kinetic model with the first-order reaction rate constant (*k*), C_t_ = C_o_ × e^−kt^, where C_t_ is the concentration of MB at time t (mg/L), and C_o_ is the initial MB concentration (mg/L).

The solid lines in Figure 7b are the fitting curves using the kinetic model. The fittings yield the *k* values of the photolysis (P) and photocatalytic processes using the S1–S6 materials (see Figure 7c). The *k* of the photolysis reaction was a small value of 1.8 × 10^−3^ min^−1^, while the *k* values were 32.1 × 10^−3^ min^−1^ (S1), 55.2 × 10^−3^ min^−1^ (S2), 38.5 × 10^−3^ min^−1^ (S3), 37.5 × 10^−3^ min^−1^ (S4), 28.9 × 10^−3^ min^−1^ (S5), 18.7 × 10^−3^ min^−1^ (S6). This indicates that MB degradation is much faster and more effective by using the photocatalysts as compared to the photolysis process. In addition, the composites of TiO_2_/AC (S2, S3, S4) exhibited an enhancement in the photocatalyst activity over either the pristine TiO_2_ (S1) or AC (S6). Among the investigated TiO_2_/AC composites with various mass mixing ratios (S2–S5), S2 possessed the highest photocatalyst activity, suggesting that TiO_2_/AC = 4:1 is the optimal mass mixing ratio between TiO_2_ P25 and micron- and sub-micron AC. Further increased AC and decreased TiO_2_ contents to TiO_2_/AC = 3:2 and TiO_2_/AC = 2:3 also exhibited higher photocatalytic activities than that for TiO_2_. Meanwhile, the sufficient low TiO_2_ and high AC contents for the TiO_2_/AC = 1:4 (S5) lead to a decrease in activity as compared to pristine TiO_2_ (see the dashed line in Figure 7c). Noticeably, the observed decrease in MB concentration over t for AC (S6) material is attributed to the excellent adsorption characteristic of porous carbon materials (Figure 7c inset) [29,38,39].

AC/TiO_2_ composites with mass mixing ratios of (4:1), (3:2), and (2:3) have higher MB removal performance than either TiO_2_ or AC, which is attributed to the synergistic effect of the high adsorption capability of AC and the high photocatalytic activity of TiO_2_. As illustrated in Figure 7d, under UV-VIS irradiation, electron/hole (e^−^/h^+^) pairs are generated, which lead subsequently to oxidation and reduction reactions in the treated solution to generate highly active free radicals, primarily ^•^OH and ^•^O_2_^−^ (Figure 7d) [29,38,39], which in turn degrade MB dye. It is well-known that the recombination rate of e^−^/h^+^ in TiO_2_ is fast. Meanwhile, in TiO_2_/AC composites, the photogenerated carriers can be transferred between TiO_2_ and AC, allowing an increase in e^−^/h^+^ separation, reducing the e^−^/h^+^ recombination rate, and consequently enhancing the photocatalytic activity. The AC (S6) removes MB with *k* of 18.7 × 10^−3^ min^−1^ (58.3% of *k* for TiO_2_), suggesting the AC has high adsorption capacity with many sufficient active adsorption sites and a large surface area [29,38,39]. The lower *k* value of S5 than S1 indicates that a composite with too little TiO_2_ and too much AC contents (TiO_2_/AC = 1:4 for the present case) will not give an enhancement of the photocatalytic activity. An advantage of a combination of a good photocatalyst with a good adsorption material (i.e., TiO_2_/AC) is that TiO_2_ degrades MB to create renewable active adsorption sites on the AC to adsorb more MB molecules, as evidenced by the higher adsorption efficiency of pollutants near TiO_2_ positions on the composite surfaces [29,40]. Also, TiO_2_ plays a role in the photocatalytic regeneration of AC, which allows for minimizing operational costs and product waste. The optimal TiO_2_/AC (S2) obtained a high MB removal efficiency of 96.6% after 60 min of treatment at the initial MB concentration of 10 mg/L and a composite dosage of 333.3 mg/L. This efficiency is comparable to that for the TiO_2_/AC (i.e., 86.5%, 97.1%, and 99.4%, depending on the type of AC) under similar experimental conditions (i.e., reaction time of 60 min, UV light source, catalyst dosage of 400 mg/L, and MB initial concentration of 20 mg/L) [29].

## 4. Conclusions

In this study, TiO_2_/AC composites with various mass mixing ratios were prepared through a facile process. The composites exhibited tight decoration of TiO_2_ nanoparticles on micron-/submicron AC particles. TiO_2_ had crystal phases of dominant anatase and minor rutile, and the crystallite size of TiO_2_ was ~21 nm. Meanwhile, AC presented the XRD peaks of graphite and carbon, and it had a crystallite size of ~49 nm. TiO_2_/AC composites are reasonably composed of the main elements (O, Ti, C). The EDS mapping confirmed the uniform distribution of elements, indicating the formation of uniform TiO_2_/AC composites. Among the TiO_2_/AC composites prepared at different mixing ratios of (4:1), (3:2), (2:3), (1:4), bare TiO_2_, and bare AC, the TiO_2_/AC = 4:1 possessed the highest photocatalytic activity in degradation of MB under UV-Vis irradiation, which yielded a high MB removal efficiency of 96.6% after 60 min treatment. The enhanced photocatalytic activity of TiO_2_/AC composites is attributed to the synergistic effect of the high adsorption capability of AC and the high photocatalytic activity of TiO_2_. In addition, TiO_2_/AC composites allow charge transfer for enhanced e^−^/h^+^ separation to reduce their recombination rate and enhance their photocatalytic activity. The TiO_2_/AC composite, with an optimal weight ratio of 4:1, is suggested for treating industrial or household wastewater containing organic pollutants.

## Data Availability

The original contributions presented in the study are included in the article, further inquiries can be directed to the corresponding authors.
